# Halophyte *Nitraria billardieri CIPK25* promotes photosynthesis in *Arabidopsis* under salt stress

**DOI:** 10.3389/fpls.2022.1052463

**Published:** 2022-12-16

**Authors:** Lu Lu, Xinru Wu, Yao Tang, Liming Zhu, Zhaodong Hao, Jingbo Zhang, Xinle Li, Jisen Shi, Jinhui Chen, Tielong Cheng

**Affiliations:** ^1^ Key Laboratory of Forest Genetics & Biotechnology of Ministry of Education of China, Co-Innovation Center for Sustainable Forestry in Southern China, Nanjing Forestry University, Nanjing, China; ^2^ Experimental Center of Desert Forestry, Chinese Academy of Forestry, Dengkou, Inner Mongolia, China; ^3^ College of Biology and the Environment, Nanjing Forestry University, Nanjing, China

**Keywords:** halophyte, *Nitraria billardieri*, *CIPK25*, salt adaptability, photosynthesis

## Abstract

The calcineurin B-like (CBL)-interacting protein kinases (CIPKs), a type of plant-specific genes in the calcium signaling pathway, function in response to adverse environments. However, few halophyte derived CIPKs have been studied for their role in plant physiological and developmental adaptation during abiotic stresses, which inhibits the potential application of these genes to improve environmental adaptability of glycophytes. In this study, we constructed *Nitraria billardieri CIPK25* overexpressing *Arabidopsis* and analyzed the seedling development under salt treatment. Our results show that *Arabidopsis* with *NbCIPK25* expression exhibits more vigorous growth than wild type plants under salt condition. To gain insight into the molecular mechanisms underlying salt tolerance, we profiled the transcriptome of WT and transgenic plants *via* RNA-seq. GO and KEGG analyses revealed that upregulated genes in *NbCIPK25* overexpressing seedlings under salt stress are enriched in photosynthesis related terms; Calvin-cycle genes including glyceraldehyde-3-phosphate dehydrogenases (GAPDHs) are significantly upregulated in transgenic plants, which is consistent with a decreased level of NADPH (GAPDH substrate) and increased level of NADP^+^. Accordingly, *NbCIPK25* overexpressing plants exhibited more efficient photosynthesis; soluble sugar and proteins, as photosynthesis products, showed a higher accumulation in transgenic plants. These results provide molecular insight into how *NbCIPK25* promotes the expression of genes involved in photosynthesis, thereby maintaining plant growth under salt stress. Our finding supports the potential application of halophyte-derived *NbCIPK25* in genetic modification for better salt adaptation.

## Introduction

Soil salinity is highly detrimental to plants because it alters plant structure and limits plant growth (Munns and Tester, 2008). Soil salinization increases the barrier to address food shortages and ecological damage in addition to accelerating climate change (Tester and Langridge, 2010). Plants mainly face two kinds of pressure from salt stress: osmotic stress and ionic stress (Munns and Tester, 2008). Osmotic stress results from soil salinity changes to osmotic pressure by reduced water availability for plants. Ionic stress then results from salt containing ions that are taken up and accumulated in plant tissues, leading to metabolic disorders and nutritional imbalance, which usually reduces growth of salt-sensitive species. On the contrary, salt-tolerant halophytes could maintain their growth and development under ionic pressure (Liang et al., 2018).

Unfortunately, most plant species are glycophytes with a limited tolerance to salinity. Water shortage and ionic accumulation could damage their cellular structures and inhibit their photosynthesis rates. For example, cotton exposed to salinity shows a distortion of its chlorophyll structure and a considerable decline in chlorophyll, consequently leading to a decreased photosynthesis rate (Zhang et al., 2014). Therefore, to promoting molecular breeding of new cultivars with increased salt tolerance, it is critical to elucidate the physiological and molecular mechanism of salinity tolerance in plants.

To cope with the adverse effects of salt stress, plants have evolved a range of species-specific mechanisms (Gautam et al., 2022). Salt-induced elevation of Ca^2+^ in cytoplasm is perceived by Ca^2+^ sensors to initiate stress signaling transduction. As one major class of players in the Ca^2+^ signaling pathway, calcineurin B-like (CBL) proteins specifically activate CBL-interacting protein kinases (CIPKs) to regulate cellular responses to various stresses (Gong et al., 2004; Luan, 2009; Sheng et al., 2009; Liu et al., 2013; Tang et al., 2015). The CIPKs contain a unique C-terminal regulatory region with a conserved NAF/FISL motif for interacting with CBLs, and a highly conserved N-terminal kinase domain with catalytic activity for phosphorylating downstream proteins (Kudla et al., 1999; Kim, 2003; Albrecht, 2014).

Many physiological responses to abiotic stresses have been assigned to CIPK function, including regulating ion balance and osmotic pressure (Guo et al., 2001; Xu et al., 2006; Ho et al., 2009; Tripathi et al., 2010; Liu et al., 2013; Zhou et al., 2014; Hu et al., 2015; Yin et al., 2020). AtCIPK24 (also known as salt overly sensitive 2, SOS2) activates the plasma membrane Na^+^/H^+^ antiporter SOS1 by phosphorylation, which transports Na^+^ out of cells when activated (Qiu et al., 2002; Quintero et al., 2011). A similar function has also been reported for AtCIPK8, which activates SOS1 to facilitate Na^+^ extrusion, promoting plant adaptation to saline soil (Yin et al., 2020). AtCIPK1 and AtCIPK9 are required for root K^+^ uptake to maintain plant growth (Lara et al., 2020). In addition, cotton GhCIPK6 has been reported to promote sugar accumulation, which works as main energy source for cellular activities, as well as cellular solutes for osmotic balance (Tognetti et al., 2013; Deng et al., 2020). The overexpression of *MdCIPK22* from apple promotes sugar accumulation and improves drought tolerance of transgenic plants (Ma et al., 2019). These results reveal the multifaceted regulation of CIPK kinases on various abiotic stresses.

To date, most plants that have been tested experimentally to elucidate the mechanisms of salt tolerance are glycophytes. Unlike halophytes, glycophytes have relatively poor resistance to salt stress and provide a limited number of functional genes that may improve salt tolerance (Flowers and Colmer, 2010; Cheeseman, 2014). *Nitraria billardieri* (*N. billardieri*) is a typical halophyte, growing in arid and saline soil, which is routinely utilized to stabilize sand deposits and reduce soil salt content (Zhang et al., 2015). Due to their unique physiological characteristics that allow them to grow on saline soils, *Nitraria* species are ideal plants for studying the mechanisms of salt tolerance (Li et al., 2021). Therefore, we used *N. billardieri* to identify genes that are involved in salt tolerance and isolated *NbCIPK25* from this halophyte, which gene was found to positively respond to salt stress (Lu et al., 2022). To explore the molecular mechanism of *NbCIPK25* function in salt response, we overexpressed *NbCIPK25* in *Arabidopsis* and found it affects the expression of different genes under normal or salt condition. Our results reveal the positive function of *NbCIPK25* overexpression on salt adaptability of *Arabidopsis*, thus suggesting a potential application of this halophyte derived gene in increasing the salt tolerance of other glycophyte species.

## Materials and methods

### Plant materials and treatments

The *Arabidopsis thaliana* (*A. thaliana*) Columbia ecotype was used to produce *NbCIPK25* transgenic plants. *NbCIPK25* gene was isolated from *N. billardieri* leaf and expressed under the control of the cauliflower mosaic virus (CaMV) 35S promoter in the pBI121 binary vector with a selectable marker of neomycin phosphotransferase II (NPT II). The pBI121-*NbCIPK25* vector was transformed into *Agrobacterium tumefaciens* GV3101 for plant transformation through the floral dip method (Clough and Bent, 1998). Positive transgenic plants were selected using 50 mg/L Kanamycin and confirmed by target gene PCR product sequencing. Semi-quantitative PCR (semi-qPCR) has been used to evaluate *NbCIPK25* overexpression in transgenic plants; *Arabidopsis UBIQUITIN 10* (*AtUBQ10*) was taken as reference; primers used in this experiment is listed in **Supplementary Table 1**.

*NbCIPK25* homozygous transgenic seeds were used for phenotypic observation and physiological analysis. *Arabidopsis* seeds were surface sterilized with 1% sodium hypochlorite for 15 min, washed with sterile water and sown on ½-strength Murashige-Skoog (½MS) medium containing different concentrations of NaCl for seed germination analysis. The data was collected 5 days after germination, with three biological replicates. A one-way ANOVA test was performed using IBM SPSS Statistics 23.0 for statistical analysis.

For seedling phenotype observation, we have transferred 5-day-old seedlings germinated on normal ½MS medium to fresh medium with 0 mM or 100 mM NaCl. After 7-day-treatment, the number of leaves and percentage of seedlings with leaf chlorosis were recorded. To collect dry weight data, the seedlings were oven-dried at 80 °C until constant weight.

To analyze photosynthetic activity of plants under salt stress, 5-day-old seedlings were planted on soil in pots. After 4 weeks, the plants were irrigated with 0 mM or 200 mM NaCl for 24 h, after which photosynthetic efficiency was measured by chlorophyll fluorescence analysis. Plants treated for 10 days with salt water were harvested to test soluble sugar and protein content in leaves using three biological replicates. Statistical analysis was performed with t-test provided by GraphPad Prism 8 software.

### RNA-seq and bioinformatic analysis

5-day-old wild-type (WT) and *NbCIPK25*-overexpressing seedlings that were treated with 0 mM NaCl or 100 mM NaCl for 6 h were harvested for RNA sequencing. Total RNA was isolated using the Eastep^®^ Super Total RNA Purification Kit (Promega, Shanghai, China), after which sequencing was conducted with an Illumina HiSeq platform, generating 150-bp paired-end reads. Clean reads that were used for further analyses were obtained by removing low-quality reads or those containing an adapter and poly-N. Paired-end clean reads were aligned to the *A. thaliana* reference genome using Hisat2 v2.0.4 (Kim et al., 2015). Read mapping was accomplished with HTSeq v0.9.1. Genes with a |log2fold| > 0.5 and an adjusted *p*-value (*q*-value) < 0.05 were assigned as DEGs, which were analyzed using the DESeq2 R package (Anders and Huber, 2010). The fragments per kilobase of transcript per million mapped reads (FPKM) data (Trapnell et al., 2010) for each DEG was used to construct expression heatmaps. We used clusterProfiler R package to implement PCA analysis, Gene Ontology (GO) and Kyoto Encyclopedia of Genes and Genomes (KEGG) pathways enrichment analysis of DEGs. GO terms with a corrected *p* value less than 0.05 were considered significantly enriched in DEGs.

### Quantitative real-time PCR analyses

To confirm the expression level of genes involved in photosynthesis, quantitative real-time PCR (qPCR) analysis was performed using total RNA extracted from *NbCIPK25* transgenic plants treated with 0 or 100 mM NaCl for 6 h. Total RNA was reverse transcribed following the manufacturer’s instruction of the HiScript III 1st Strand cDNA Synthesis Kit (+gDNA wiper) (Vazyme Biotech, Nanjing, China). The qPCR was carried out using TB Green^®^ Premix Ex Taq™ (Takara, Dalian, China) and a LightCycler^®^480 qPCR detection system (Roche, Basel, Switzerland). The expression of target genes was normalized by the expression level of the housekeeping gene *UBQ10* in *Arabidopsis* (Norris et al., 1993). Three biological and experimental repeats were performed. The accession numbers of genes tested by qPCR and the primers are listed in **Supplementary Table 1**.

### NADPH, NADP^+^, soluble sugar and protein content measurement

NADPH and NADP^+^ were quantified with kits purchased from Sangon Biotech (D799249-0050, Shanghai, China) following the user manual. Soluble sugar content was tested using the “Plant soluble sugar content test kit” from Nanjing Jiancheng Bioengineering Institute (A145-1-1, Nanjing, China). Soluble protein was measured using Coomassie Brilliant Blue dyes G250 with bovine serum albumin as a standard (Girija et al., 2020).

### Determination of chlorophyll fluorescence parameters

Chlorophyll fluorescence and P700 absorption changes in the PSI reaction center were tested simultaneously for WT and transgenic plants using a portable chlorophyll fluorometer Dual-PAM-100 Chlorophyll Fluorometer (Waltz) (Basso et al., 2020). The experimental plants were moved into a dark room for 20-60 min for dark adaptation. The measurement was conducted on the 3rd leaf counting from the top downward on plants under normal and salt conditions. The chlorophyll concentration was measured according to a previously reported study (Lichtenthaler and Wellburn, 1983).

## Results

### *NbCIPK25* promotes seedling growth under salt stress

**Figure 1:**
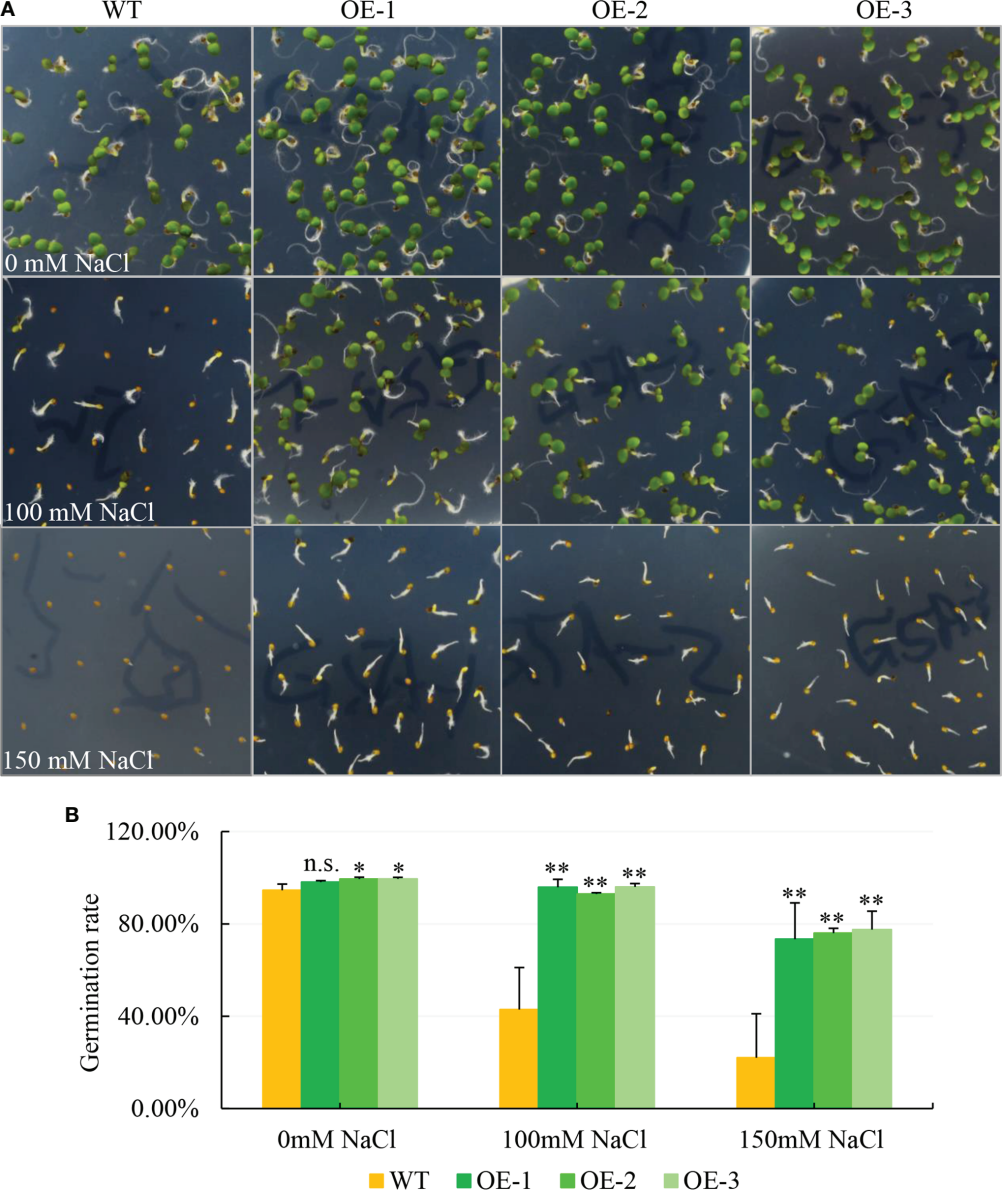
*NbCIPK25* overexpression promotes the germination rate and growth of *Arabidopsis* on salt medium. **(A)** The phenotype of seed germination under various salt condition. WT mean wild type Col *Arabidopsis*. OE-1, 2, 3 represent three independent transgenic lines of *NbCIPK25* overexpression. 180 seeds in total per line were used in this experiment. The seeds sterilized with 1% sodium hypochlorite for 15 min were planted on 1/2 MS containing 0, 100 or 150 mM NaCl, respectively. **(B)** Statistical analysis for the germination rate of WT and transgenic seeds grown under the indicated NaCl concentration. Data represent means ± standard deviation (SD) from three biological replicates, statistics was analyzed by one-way ANOVA test, ‘*’ *p* < 0.05, ‘**’*p* < 0.01, n.s., not significant.

To explore the function of the halophyte-derived gene *NbCIPK25* (NCBI accession number: MZ353017), we overexpressed this gene in *Arabidopsis*, which was verified by semi-qPCR result as a relatively high expression of *NbCIPK25* in transgenic plants (**Supplementary Figure 1A**). To test whether OE-*NbCIPK25* affects germination rate, we germinated the seeds of OE-*NbCIPK25* and WT on ½ MS medium containing different concentrations of NaCl. We found that the germination rate of transgenic seeds was higher than WT under normal condition, and that this difference was significantly greater under 100 mM NaCl and 150 mM NaCl treatment (**Figures 1A, B**). Notably, OE-*NbCIPK25* seedlings grew better than WT seedlings germinated on medium with 100 mM NaCl (**Figure 1A**). Both the WT and transgenic seedlings exhibited a severe delay in leaf and root growth under 150 mM NaCl treatment compared with normal growth medium (**Figure 1A**). These results revealed a positive role of *NbCIPK25* in seed germination and seedling growth under moderate salt condition.

**Figure 2:**
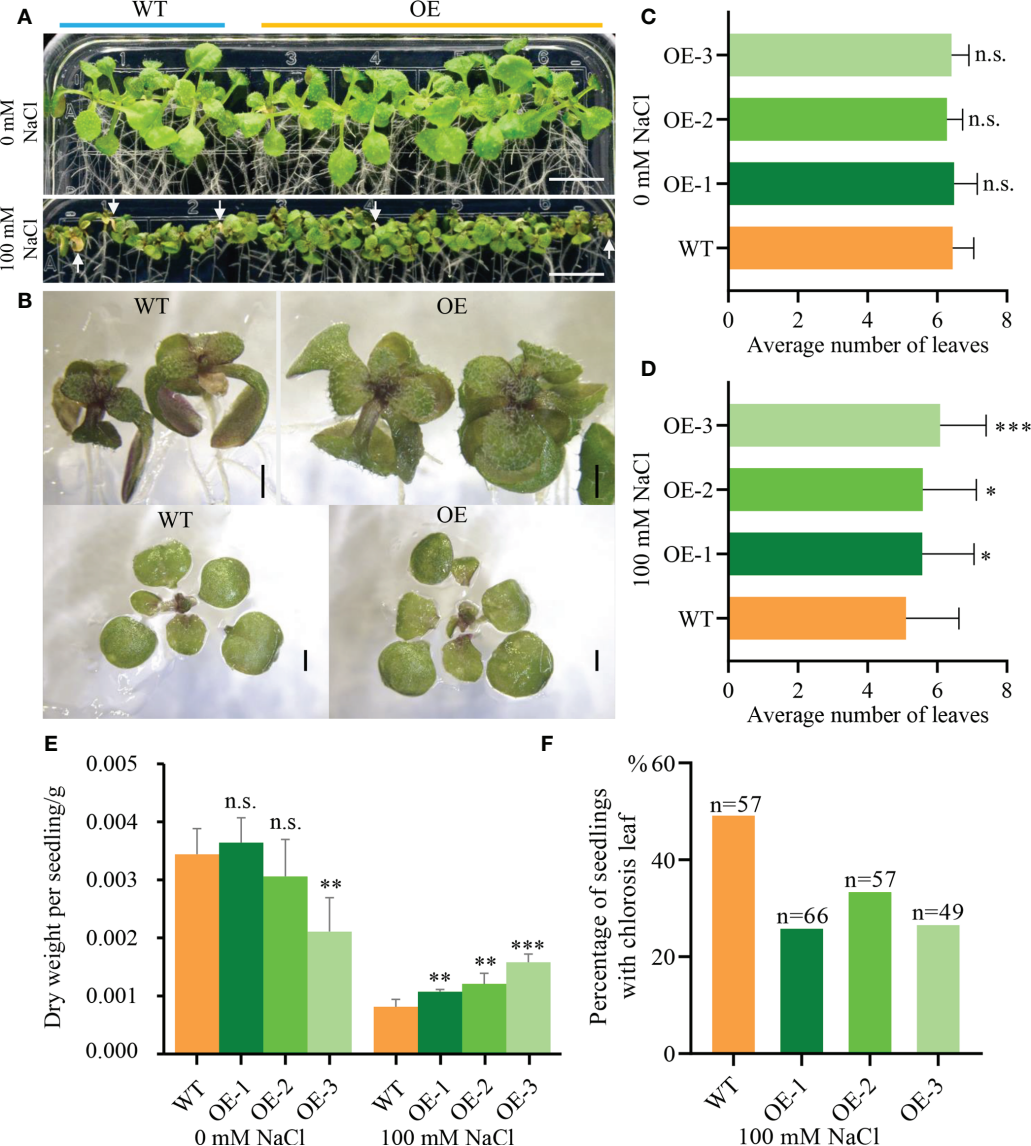
*NbCIPK25* overexpression maintains the *Arabidopsis* growth under salt condition. **(A)** The phenotype of 5-day-old WT and *NbCIPK25* overexpressing plants treated with 0 mM NaCl or 100 mM NaCl (showing at left side of panel **A**) for 7 days. Seedlings covered with blue line came from wild type (showing as WT) seeds. Seedlings under orange line were from transgenic seeds. OE represents *NbCIPK25* overexpressing plants. The white arrow heads indicate chlorotic leaves. Scale bar: 1 cm. **(B)** Shoots and leaves of WT and *NbCIPK25* transgenic plants treated with 100 mM NaCl for 7 days. Scale bar: 0.1 cm. **(C–F)** Statistical analysis for leaf number **(C, D)**, dry weight **(E)**, and percentage of seedlings with chlorotic leaves **(F)** in WT and transgenic plants treated with or without salt treatment. Data represent means ± SD from three biological replicates, statistics was analyzed by t-test using GraphPad Prism 8, ‘*’ *p* < 0.05, ‘**’*p* < 0.01, ‘***’*p* < 0.001, n.s., not significant; n, the number of examined seedlings.

To further analyze how *NbCIPK25* influences plant growth under salt stress, we germinated seeds under normal condition for 5 days, then transferred the seedlings to medium with or without salt and tracked their growth. The results showed that salt treatment inhibits seedling growth of both WT and transgenic plants in comparison to seedlings grown under normal condition (**Figure 2A**). By contrast, transgenic plants showed a more vigorous growth under salt stress than WT plants (**Figures 2A, B**). The average number of leaves grown by transgenic seedlings was significantly higher than that grown by WT seedlings under salt condition (**Figure 2D**), with no significant difference observed under normal condition (**Figure 2C**). In addition, while transgenic plants accumulated a lower dry weight than WT plants under normal growth condition, under salt stress, they accumulated a significantly higher weight (**Figure 2E**). We analyzed the percentage of seedlings with leaf chlorosis and found that WT seedlings suffering from salt stress showed a higher rate of leaf chlorosis than transgenic seedlings (**Figure 2F**). Based on these results, we conclude that *NbCIPK25* overexpression decreases salt stress inflicted growth retardation in seedlings.

### *NbCIPK25* overexpression induces specific gene expression under salt stress

**Figure 3:**
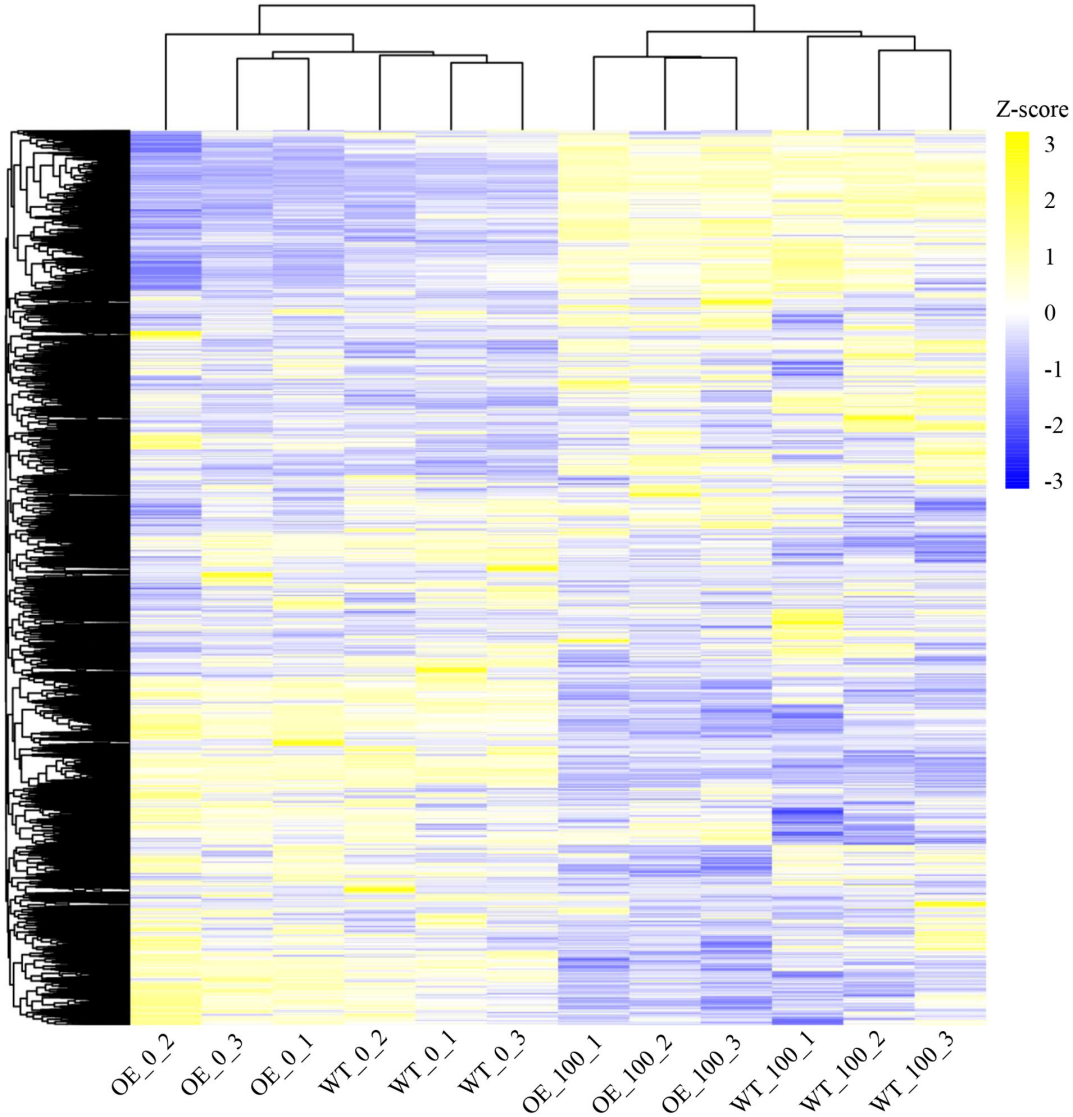
Different response of genes to salt stress between WT and *NbCIPK25* transgenic plants. Heat map representation for all genes identified in the transcriptome shown as colored boxes along with corresponding data for fragments per kilobase of transcript per million mapped reads (FPKM). Z-score is used to evaluate the relative expression level of genes in rows. Blue in color-coded boxes indicates a relative low FPKM value, and yellow indicates a relative high FPKM value in rows. OE_0 and OE_100 represent *NbCIPK25* transgenic plants under 0 or 100 mM NaCl treatment, respectively; WT_0 and WT_100 mean WT plants under 0 or 100 mM NaCl treatment, respectively.

To investigate the molecular mechanism by which *NbCIPK25* affects seedling growth, we analyzed the transcriptomes of salt-stressed (100 mM NaCl) WT and transgenic plants *via* RNA-Seq. High readcount number of *NbCIPK25* in transgenic plants further verify the gene overexpression (**Supplementary Figure 1B**). Hierarchal clustering of total genes expressing across all samples showed that plants undergoing the same salt treatment clustered together (**Figure 3**). Under normal condition, the transcriptome of *NbCIPK25* transgenic samples is distinct from that of WT, but they have not been completely separated; under 0 mM NaCl condition, two out of three transgenic biological repeats clustered together with three WT samples (**Figure 3**). Under salt treatment, the three transgenic replicates clustered together, separate from the three WT replicates (**Figure 3**).

**Figure 4:**
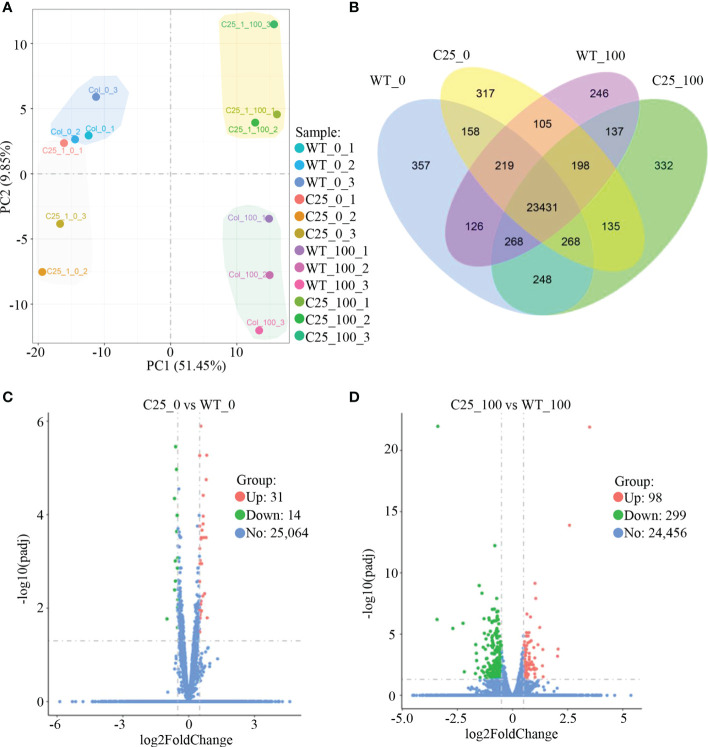
*NbCIPK25* induces differential expression of genes under salt stress. **(A)** PCA analysis of the allele expression in wild type (showing as WT) and transgenic seedlings (showing as C25) under 0 or 100 mM NaCl treatment with ggplot2 in R (Version 3.0.3). WT_0 and C25_0 means no salt treatment, WT_100 and C25_100 represent plants treated with 100 mM NaCl. **(B)** Venn diagram of expressed genes in the genotypes as indicated. Blue shading means genes expressed in WT under normal condition; yellow shading represents genes expressed in transgenic plants under normal condition; purple shading indicates genes expressed in WT under 100 mM NaCl treatment; green shading contains genes expressed in transgenic plants under 100 mM NaCl treatment. **(C, D)** Volcano plots of fold changes of differentially expressed genes (DEGs) from *NbCIPK25* overexpressing plants vs WT under the condition with or without salt treatment. Red and green dots represent upregulated and downregulated DEGs, respectively. |Log2Foldchange| >0.5 and adjust *p* value (*q* value) < 0.05 were set as cut-off value to screen DEGs by DESeq2 with R package. “31” and “14” in panel C mean the numbers of upregulated and downregulated DEGs from transgenic plants vs WT under normal condition, respectively. “98” and “299” mean the numbers of upregulated and downregulated DEGs from transgenic plants vs WT under salt condition, respectively.

Consistently, a principal component analysis (PCA) similarly showed a close relationship among plants grown under normal condition, but a clear separation between WT and transgenic plants under salt stress (**Figure 4A**). Our results were further confirmed by a gene expression classification visualized in a Venn diagram (**Figure 4B**). 23,431 genes are expressed in all samples. Under 100 mM NaCl treatment, 332 genes are uniquely expressed in *NbCIPK25* overexpression lines (**Figure 4B**). We used |log2fold| > 0.5 and an adjusted *p*-value (*q*-value) < 0.05 as cutoff to identify 98 upregulated genes and 299 downregulated genes as differentially expressed genes (DEGs) between transgenic and WT plants under 100 mM NaCl treatment (**Figure 4D**). By contrast, we identified 31 upregulated and 14 downregulated genes as DEGs between transgenic and WT plants grown under normal condition (**Figure 4C**). Moreover, we identified 3,094 DEGs between WT under 100 mM NaCl treatment and WT under 0 mM NaCl treatment (**Supplementary Figure 2A**), which number is 4,529 between transgenic plants under 100 mM NaCl treatment and transgenic plants under 0 mM NaCl treatment (**Supplementary Figure 2B**); 1108 upregulated DEGs and 1,552 downregulated DEGs specifically exist in *NbCIPK25* overexpressing plants treated with salt stress (**Supplementary Figure 2C**). These results show that *NbCIPK25* overexpression causes differential gene expression in comparison to WT under salt treatment.

### *NbCIPK25* affects photosynthesis in *Arabidopsis* under salt stress

**Figure 5:**
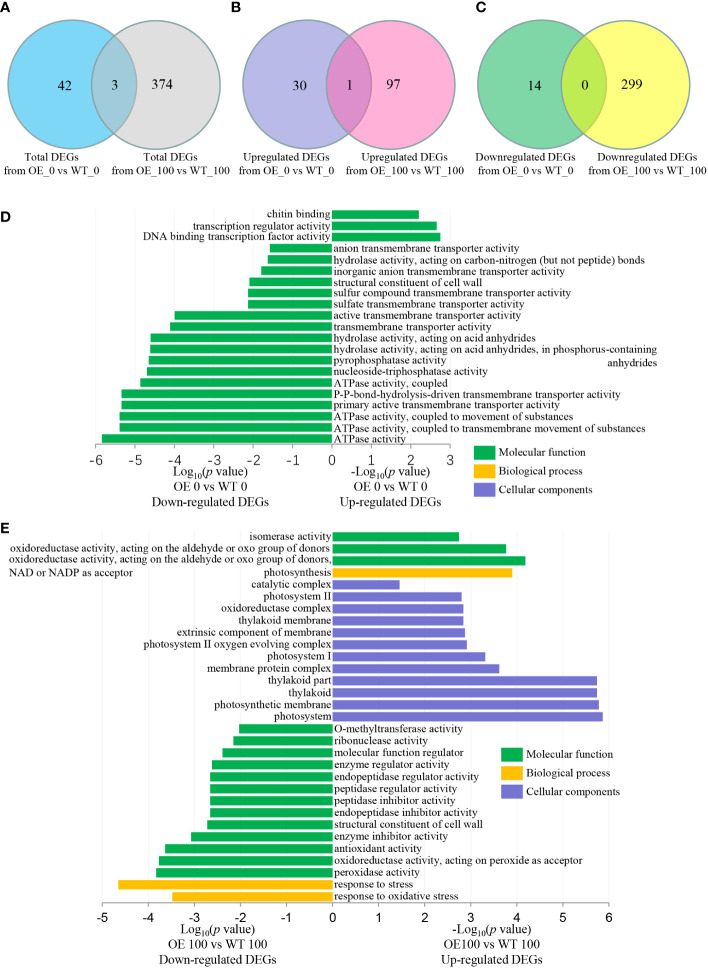
*NbCIPK25* induces DEGs enriched for different GO terms between normal and salt condition. **(A–C)** Venn diagram of total **(A)**, upregulated **(B)** and downregulated **(C)** DEGs from *NbCIPK25* overexpressing plants vs WT under 0 mM NaCl or 100 mM NaCl treatment. “3” in panel A means the overlapping DEG number between total DEGs caused by *NbCIPK25* overexpression under normal condition and salt condition. “1” in panel B means the overlapping DEG number between upregulated DEGs caused by *NbCIPK25* overexpression under normal condition and salt condition. “0” in panel C means no overlapping DEG between downregulated DEGs caused by *NbCIPK25* overexpression under normal condition and salt condition. **(D, E)** Functional classification of upregulated and downregulated DEGs from *NbCIPK25* overexpressing plants vs WT under 0 mM NaCl treatment **(D)** and 100 mM NaCl treatment **(E)**, respectively, using GO annotations with *p* < 0.05 as cut-off value.

CIPKs have been previously reported to function in calcium signaling pathways that respond to abiotic stresses through various molecular and physiological mechanisms (Ma et al., 2021). To understand why transgenic seedlings show faster growth, we performed GO analysis with clusterProfiler. The DEGs induced by *NbCIPK25* overexpression under normal conditions showed little overlap with those DEGs induced under salt stress (**Figure 5A**); only a single common gene was identified between the two groups of upregulated DEGs (**Figure 5B**), while no common genes were shared between the two groups of downregulated DEGs (**Figure 5C**). Under normal condition, upregulated DEGs from transgenic plants vs WT were significantly enriched in GO terms related to transcription factor activity and chitin binding (**Figure 5D**), while downregulated DEGs were enriched in 21 GO terms describing molecular functions such as ATPase activity, hydrolase activity, and transmembrane transporter activity (**Figure 5D**). By contrast, under salt treatment, *NbCIPK25* overexpression induced upregulated DEGs were significantly enriched for molecular function GO terms describing the photosynthesis biological pathway, photosystem related cellular components and oxidoreductase activity as well as isomerase activity (**Figure 5E**); downregulated DEGs were enriched for oxidoreductase activity GO terms (**Figure 5E**).

**Figure 6:**
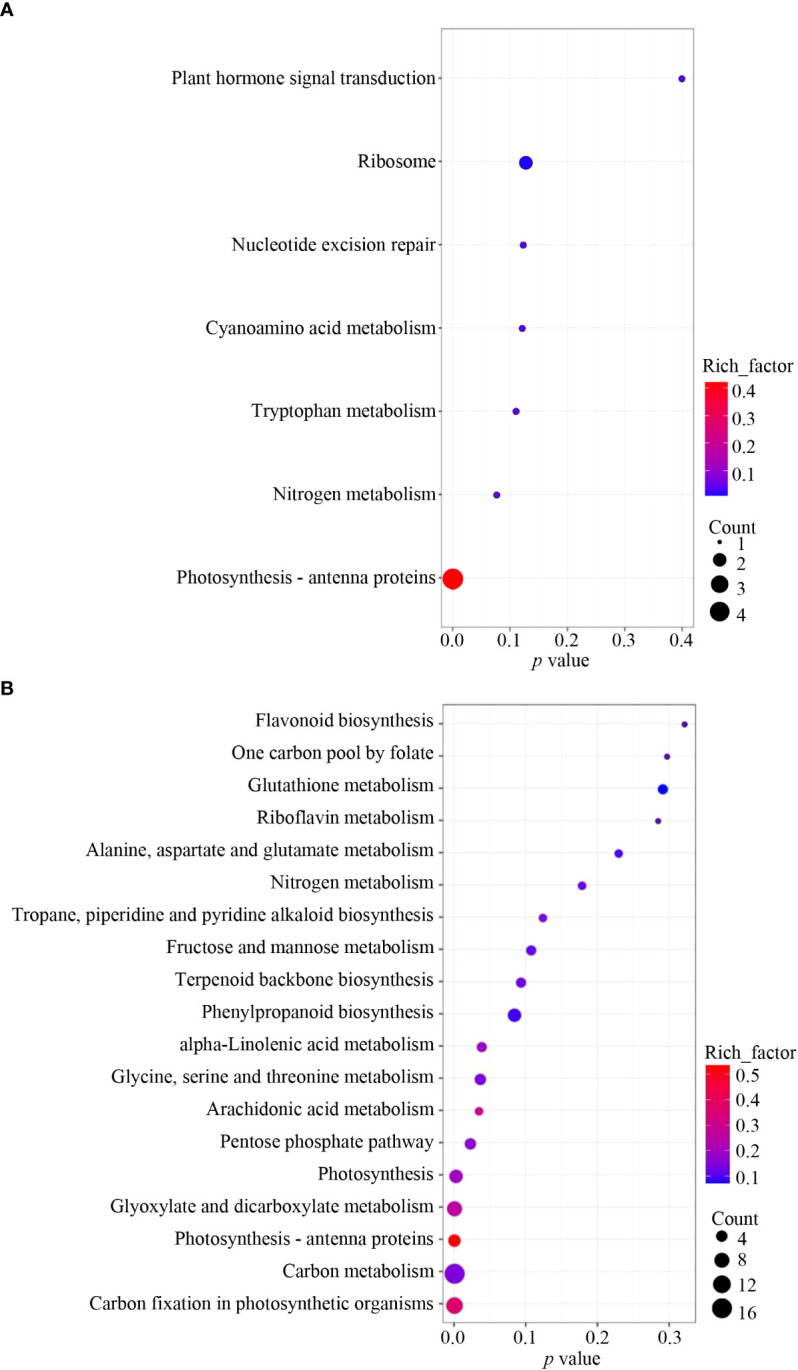
*NbCIPK25* causes DEGs involved in different KEGG pathways between normal and salt treatment. **(A, B)** KEGG pathway enrichment analysis of total DEGs induced by *NbCIPK25* overexpression under 0 mM NaCl **(A)** and 100 mM NaCl treatment **(B)**. Dot size indicates the DEG numbers. *P* values locate at the bottom of each figure.

**Figure 7:**
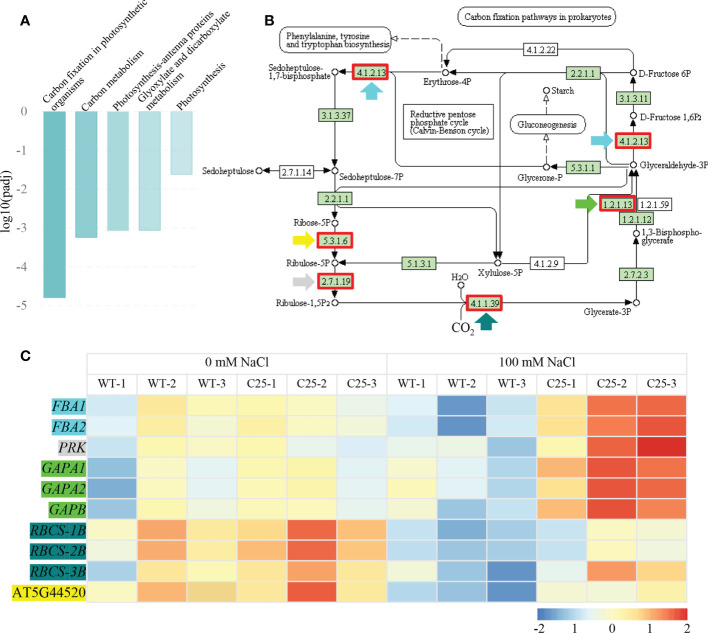
*NbCIPK25* overexpression induces DEGs involved in photosynthesis. **(A)** KEGG enrichment of upregulated DEGs caused by *NbCIPK25* overexpression under salt treatment. **(B)** The network of “Carbon fixation in photosynthetic organisms”, red boxes indicate the up-regulated DEGs. Colored arrow heads are used to mark the names of DEGs in panel C. **(C)** The transcriptional trends of DEGs involved in “Carbon fixation in photosynthetic organisms” shown as colored boxes with FPKM. Different color backgrounds under gene names correspond with colored arrow heads in **Figure 7B**, to indicate the corresponding location of genes in the pathway. FBA: Fructose-bisphosphate aldolase; PRK, Phosphoribulokinase; GAPA, Glyceraldehyde 3-phosphate dehydrogenase; RBCS, Ribulose-1,5-bisphosphate carboxylase.

To further verify the potential pathways in which *NbCIPK25* functions, we conducted KEGG analysis with clusterProfiler. We found that only the term “Photosynthesis - antenna proteins” was significantly enriched when analyzing all DEGs from transgenic plants vs WT under normal condition (**Figure 6A**). Nevertheless, salt-induced DEGs from transgenic plants vs WT were significantly enriched for the pathways “Carbon fixation in photosynthetic organisms”, “Carbon metabolism”, “Photosynthesis - antenna proteins”, “Glyoxylate and dicarboxylate metabolism” and “Photosynthesis” (**Figure 6B**). These enriched KEGG pathways were mainly in the upregulated DEGs category; “Carbon fixation in photosynthetic organisms” (pathway ID: ath00710) was the most significantly enriched pathway (**Figure 7A**). The network showed that genes encoding key enzymes involved in carbon fixation were significantly upregulated, including Ribulose-1,5-bisphosphate carboxylases (RBCS), Glyceraldehyde 3-phosphate dehydrogenase (GAPA/GAPB), and Fructose-bisphosphate aldolase (FBA) (**Figure 7B**). Although the transcription of RBCSs in transgenic plants was higher than that of WT, these genes were downregulated in both of WT and transgenic plants under salt treatment (**Figure 7C**). On the contrary, the expression of GAPA1/2, GAPB and FBAs was further enhanced in transgenic plants under 100 mM NaCl condition (**Figure 7C**). These data indicate that *NbCIPK25* overexpression promotes photosynthesis of plants under salt stress *via* inducing the expression of genes involved in carbon fixation.

### *NbCIPK25* influences photosynthesis yield

**Figure 8:**
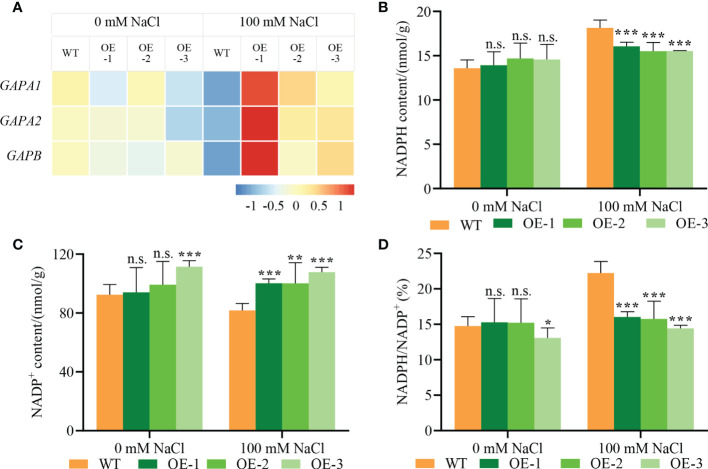
*NbCIPK25* enhances the transcription of key genes in the Calvin cycle. **(A)** The transcription trends of GAPDHs shown as colored boxes from qPCR results. *AtUBQ10* was taken as internal standard. The gene expression levels were normalized to the gene expressing in WT under 0 mM NaCl treatment for calculating foldchanges of expression levels. Colored bar with values under the panel A was used to evaluate gene expression level. Blue boxes mean downregulated expression; red boxes mean upregulated expression. **(B–D)** NADPH content **(B)**, NADP^+^ content **(C)** and NADPH/NADP^+^ **(D)** of 5-day-old WT and transgenic plants under 0 mM NaCl or 100 mM NaCl for 2 days, data represent means ± SD from three biological replicates, t-test used for statistical analysis, ‘*’ *p* < 0.05, ‘**’ *p* < 0.01, ‘***’*p* < 0.001, n.s., not significant.

The DEGs related to the Calvin cycle were significantly upregulated in transgenic plants compared with WT (**Figure 7C**). We further verified the transcriptional induction of genes encoding GAPDHs (*GAPA1*, *GAPA2* and *GAPB*) by qPCR. *NbCIPK25* overexpression significantly upregulated GAPDHs in salt-stressed plants (**Figure 8A**). In addition, as the substrate of GAPDHs, NADPH content in transgenic plants was lowered compared to WT (**Figure 8B**), while NADP^+^ content was increased (**Figure 8C**). These results reveal that *NbCIPK25* overexpression promotes the consumption of NADPH, resulting in a lower ratio of NADPH/NADP^+^ (**Figure 8D**).

**Figure 9:**
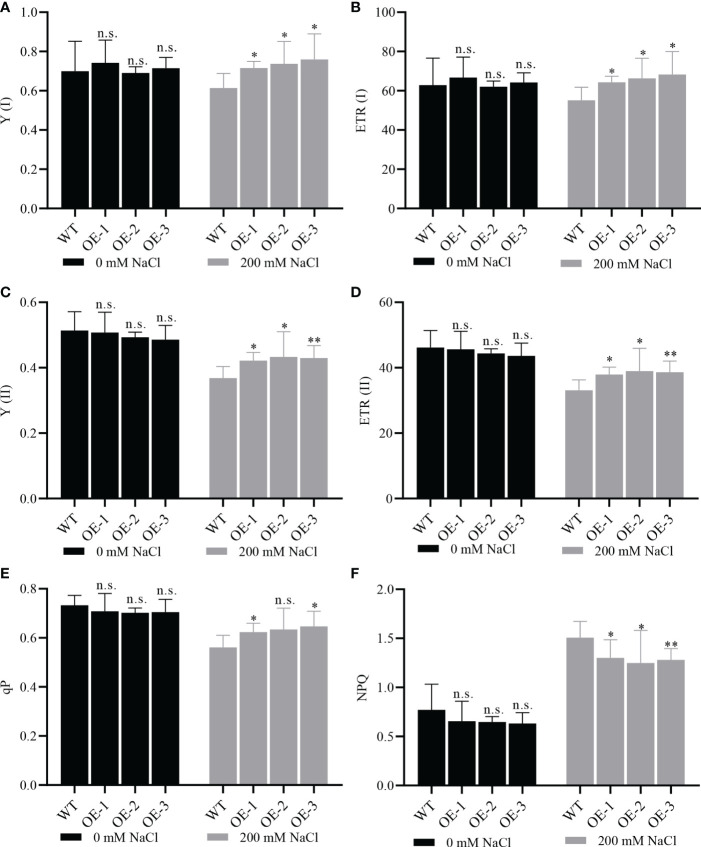
*NbCIPK25* affects photosynthesis under salt treatment. **(A–F)** Photosystem I (PSI) photochemistry, Y (I); electron transport rate of PSI, ETR(I); photosystem II (PSII) photochemistry, Y (II); electron transport rate of PSII, ETR(II); Photochemical quenching, qP; Non-photochemical quenching, NPQ, under the treatment and genotypes as indicated. WT mean wild type Col *Arabidopsis*. OE-1, 2, 3 represent three independent *NbCIPK25* transgenic lines. Data represent means ± SD from three biological replicates, t-test used for statistical analysis, ‘*’ *p* < 0.05, ‘**’ *p* < 0.01, n.s., not significant.

To study whether *NbCIPK25* overexpression enhances photosynthesis under salt stress, we utilized the Dual-PAM-100 Chlorophyll Fluorometer to measure chlorophyll fluorescence and the P700 redox state of leaves *in vivo*. Under normal conditions, we detected non-significant differences in the yield of chlorophyll fluorescence (Y (I)) and the photosynthetic electron transport rate (ETR) from PSI between WT and transgenic plants, yet under salt stress transgenic plants showed significantly higher values for these two aspects than WT (**Figures 9A, B**). A similar result was observed for the yield of chlorophyll fluorescence and photosynthetic electron transport rate from PSII (**Figures 9C, D).** The photochemical quenching parameter (qP) and non-photochemical quenching parameter (NPQ) describing the relative effects of the energy dissipation pathway exhibited a salt-induced significant difference between transgenic plants and WT, and a lower but not significant difference under normal conditions (**Figures 9E, F**). However, we found no difference in chlorophyll content between WT and transgenic plants (**Supplementary Figure 3**). These results indicate that *NbCIPK25* overexpression affects the yield of photosynthesis in *Arabidopsis* under salt stress.

### *NbCIPK25* promotes soluble sugar and protein accumulation under salt stress

**Figure 10:**
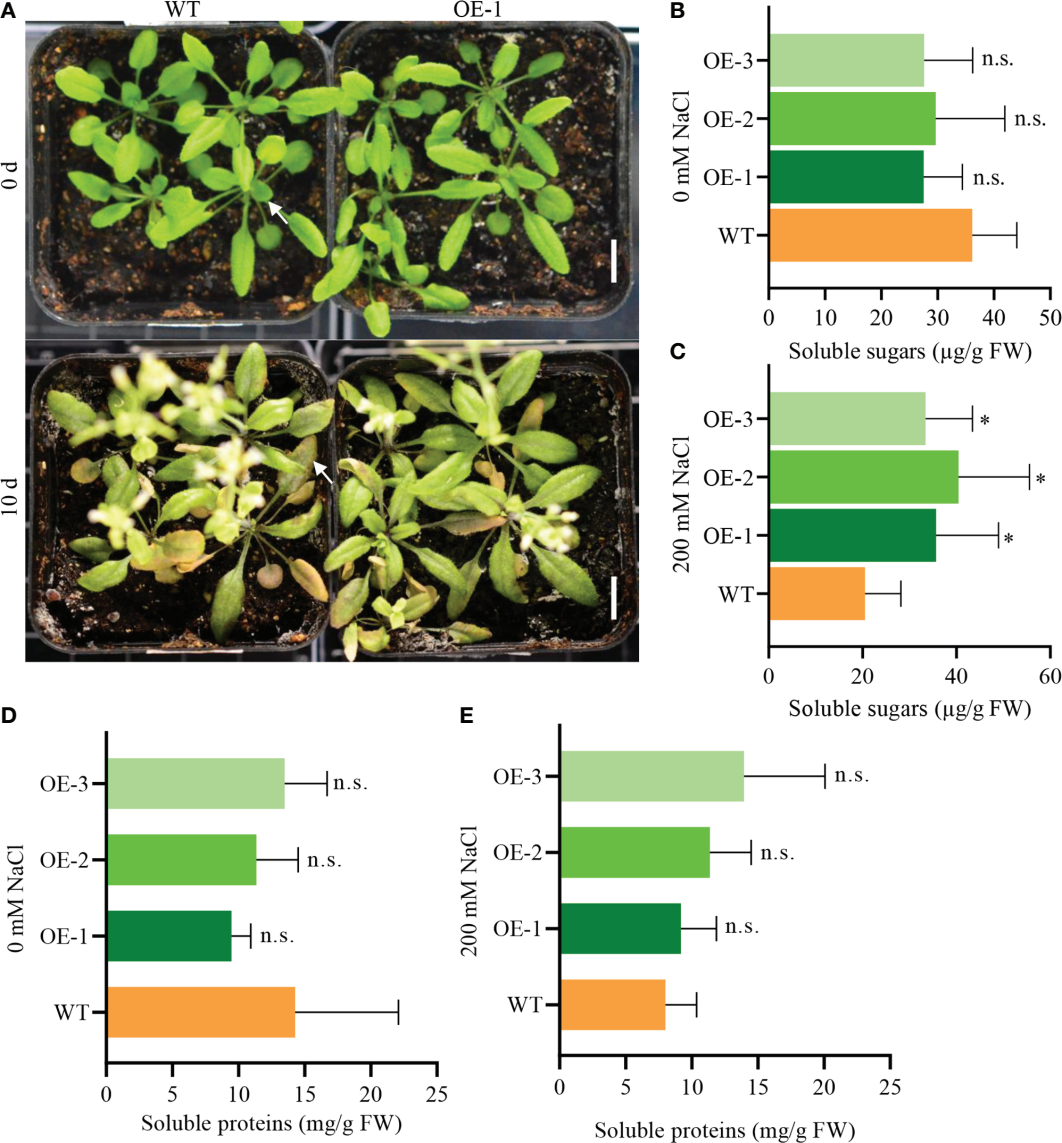
*NbCIPK25* promotes soluble sugar accumulation under salt treatment. **(A)** Phenotypes of salt-treated seedlings in pots for 0 and 10 days. Scale bar: 1 cm. **(B, C)** Soluble sugar content under normal condition **(B)** and 200 mM NaCl treatment **(C)**. **(D, E)** Soluble protein content under normal condition **(D)** and 200 mM NaCl treatment **(E)**. Data represent means ± SD from three biological replicates, t-test was used for statistical analysis, ‘*’ *p* < 0.05, n.s., not significant.

To assess photosynthesis efficiency, we measured sugar and protein levels in seedlings. Transgenic seedlings treated with 200 mM NaCl for 10 days grew healthier than WT seedlings (**Figure 10A**). The soluble sugar content in salt-stressed transgenic plants was significantly higher than in WT, but there was no difference for plants grown under normal conditions (**Figures 10B, C**). The protein content of transgenic plants was lower than WT under normal condition, but higher under salt treatment (**Figures 10D, E**). These data suggest that *NbCIPK25* promotes sugar and protein accumulation under salt stress by affecting photosynthesis, which could explain the improved growth of transgenic plants. Based on these results, we conclude that *NbCIPK25* promotes salt-adaptation of plants by enhancing the transcription of genes involved in photosynthesis, maintaining Calvin cycle function for sugar and protein production in plants under salt stress.

## Discussion

CIPKs are well known kinases in calcium signal transduction that are responsive to abiotic stresses. Based on previous studies, some CIPKs have been shown to improve plant salt tolerance by their overexpression (Hu et al., 2015; Chen et al., 2021; Li et al., 2022). However, the underlying molecular and physiological mechanisms that explain how halophyte-derived CIPKs work against salt stress are not well understood. In our previous study, we found that *CIPK25* positively responded to salt stress (500 mM NaCl) in *N. billardieri* (Lu et al., 2022). To explore the potential mechanism of a halophyte *CIPK* gene in salt adaptability, here, we overexpressed *NbCIPK25* in *Arabidopsis* and analyzed its transcriptome data under normal or salt condition.

### Salt induced different DEGs in *NbCIPK25* transgenic plants

In our study, we have observed that *NbCIPK25* overexpression affects the transcriptome under normal growth conditions, yet this does not lead to obvious changes in seedling development (**Figures 1**–**5**, **10**). However, further analysis showed that DEGs between OE-*NbCIPK25* vs WT identified under normal condition were different from those identified under salt stress (**Figures 3**, **4**, **5A**). Moreover, the number of DEGs increased after salt treatment in *NbCIPK25* transgenic plants compared with WT (**Figures 4C, D**). In addition, the GO and KEGG enrichment results revealed very few overlapping terms between these two groups of DEGs (**Figures 5D, E**, **Figure 6**). These data are highly consistent with the plant phenotype of both genotypes being very similar when growing at normal condition, but significantly different under salt treatment (**Figures 1A**, **2A**, **10A**). These results indicate that salt is likely a necessary signal for CIPKs to affect gene expression during salt stress. As upstream proteins of *CIPK* family genes, CBL proteins need to sense stress-induced calcium signal to activate CIPKs. Under normal growth condition, no calcium wave is present to stimulate CBL activities, thus leading to less cooperation between CBLs and CIPKs. On the contrary, salt triggers a calcium signaling response to initiate the reaction of calcium sensors for stress adaptability. Therefore, the changed calcium signal might be the reason for an increased number of DEGs in *NbCIPK25* overexpression plants under salt stress. Our results compare closely with those obtained from a previous study on the *HbCIPK2* gene from the halophyte *Hordeum brevisubulatum*, which also revealed an enhancement of salt tolerance by *HbCIPK2* overexpression under salt condition, but no phenotypic difference between WT and transgenic plants under normal condition (Chen et al., 2012).

### *NbCIPK25* enhances photosynthesis to accumulate sugar for salt adaptation

In general, most photosynthesis genes are repressed in transcription in response to salt stress (Kilian et al., 2007). According to our transcriptome data, most upregulated DEGs in *NbCIPK25* transgenic plants are involved in photosynthetic processes, especially in the Calvin cycle. We found that genes encoding GAPDHs and ribulose bisphosphate carboxylase (Rubisco), were expressed significantly higher in transgenic plants. However, although transgenic plants expressed the genes encoding Rubisco at a relatively higher level than WT, these same genes were repressed by salt in both WT and transgenic plants (**Figure 7C**). These results underscore the damage caused by salt on *Arabidopsis* plants, and this damage could not be reversed by the expression of *NbCIPK25*. On the contrary, the genes encoding GAPDHs were upregulated in transgenic plants but downregulated in WT under salt stress. These results suggest the potential interaction between NbCIPK25 and GAPDHs. The decreased NADPH level was consistent with the upregulation of GAPDHs. Thus, we conclude that NbCIPK25 might affect the function of GAPDHs in the Calvin cycle to maintain *Arabidopsis* photosynthesis in response to salt stress. On the other hand, we observed a higher sugar content in *NbCIPK25* transgenic plants under salt treatment (**Figure 10C**). The product of photosynthesis, sugar, is not only needed for energy storage, but also participates in response to abiotic stresses (Deng et al., 2020). It has been reported that knockdown or knockout of *GhCIPK6* in cotton causes a decreased sugar content, which could be relieved by *GhCIPK6* overexpression, suggesting a positive function of *CIPK* family genes on sugar accumulation (Deng et al., 2020). Thus, we propose that *NbCIPK25* induces DEGs in plants to regulate photosynthesis and sugar content for salt adaptation.

## Conclusion

Our study analyzed the function of *CIPK25* from halophyte *N. billardieri* on plant growth under salt conditions. We found a vigorous growth of *NbCIPK25* overexpressing plants under salt stress. Our transcriptome data revealed that *NbCIPK25* overexpression affected the expression of important genes involved in the Calvin cycle, thus leading to a higher photosynthesis yield, more sugar content and a better salt tolerance. These results suggest that *NbCIPK25* plays a positive role in salinity tolerance of plants, which suggests a possible direction for future molecular breeding programs aimed at obtaining salt resistant plants.

## Data availability statement

The datasets presented in this study can be found in online repositories. The names of the repository/repositories and accession number(s) can be found below: https://db.cngb.org/, CNP0003488.

## Author contributions

LZ and ZH carried out statistical analyses; YT, JZ and XL measured plant photosynthesis; LL and XW performed all other experiments and arranged the manuscript; JS revised the manuscript; JC and TC designed the experiments. All authors contributed to manuscript revision and approved the submitted version.
