# Supplementary material for: Halophyte Nitraria billardieri CIPK25 promotes photosynthesis in Arabidopsis under salt stress
Source: Front Plant Sci. 2022 Dec 16;13:1052463. doi: 10.3389/fpls.2022.1052463 (PMC9800929; doi:10.3389/fpls.2022.1052463)
Supplement: Supplementary file 1 [file DataSheet_1.docx]

Supplementary Material

# Supplementary Tables and Figures

**1.1 Supplementary Tables：**

| **Supplementary Table 1. Information of primers for real time-PCR analysis** | | | |
| --- | --- | --- | --- |
| **Gene name** | **Accession number** | **Primer name** | **Sequence (5’-3’)** |
| *GAPA1* | AT3G26650 | *GAPA1* F | GAAAACTCAACGGGATCGCTCT |
|  |  | *GAPA1* R | ATCGCAGACATCGAGTATACCTT |
| *GAPA2* | AT1G12900 | *GAPA2* F | AAGGAAAACTTAACGGAATTGCAT |
|  |  | *GAPA2* R | TTTTCTTGGAGACTTGCACGAC |
| *GAPB* | AT1G42970 | *GAPB* F | GGTAAACTTAACGGCATTGCACT |
|  |  | *GAPB* R | CTAAAATGCCTTTCATCGGTCCA |
| *AtUBQ10* | At4g05320.2 | *AtUBQ10* F | CCGGAAAGACCATCACCCTTG |
|  |  | *AtUBQ10* R | TGTAGTCGGCCAAAGTACGTC |
| *NbCIPK25* | MZ353017 | *NbCIPK25* F | AAGTCGGCTGGAGATACCTT |
|  |  | *NbCIPK25* R | ACAACATTGTCACCTTGCCAT |

**1.2 Supplementary Figures:**

**
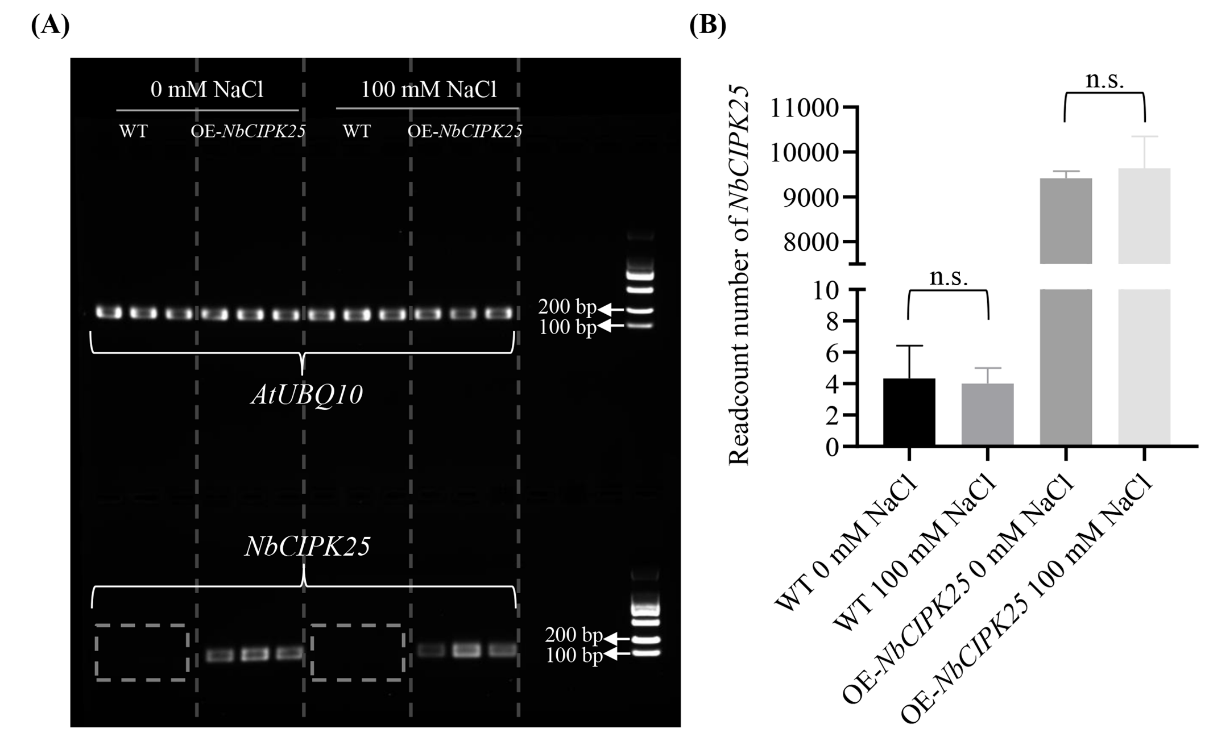
**

**Supplementary Figure 1. Transgenic plants with high transcript of *NbCIPK25*.**

**(A)** *NbCIPK25* transcript was quantified by semi-qPCR in transgenic and WT plants grown under 0 mM or 100 mM NaCl condition. *AtUBQ10* has been taken as reference gene to modify the template cDNA amount used for PCR reaction with 25 cycles. Empty area marked by dotted rectangles indicates no *NbCIPK25* production.

**(B)** *NbCIPK25* readcount number from transcriptome data of transgenic and WT plants grown under 0 mM or 100 mM NaCl condition. OE-*NbCIPK25* means *NbCIPK25* overexpression plants. n.s.: not significant by t-test.

**
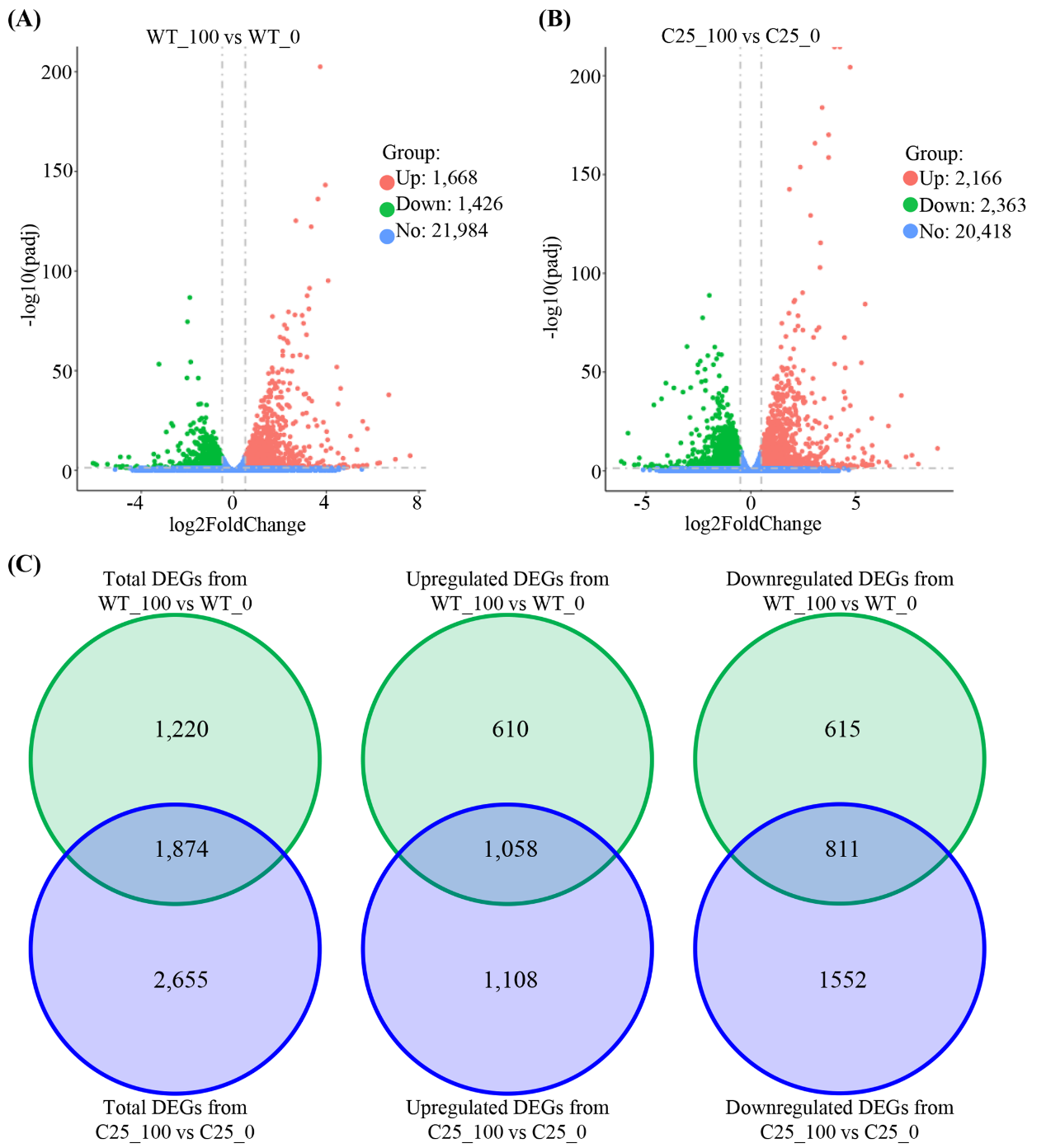
**

**Supplementary Figure 2.** ***NbCIPK25* induced differential expression of genes under salt stress.**

**(A)** DEGs identified by WT_100 vs WT_0.

**(B)** DEGs identified C25_100 vs C25_0.

**(C)** Venn diagram of DEGs identified from indicated compared groups.


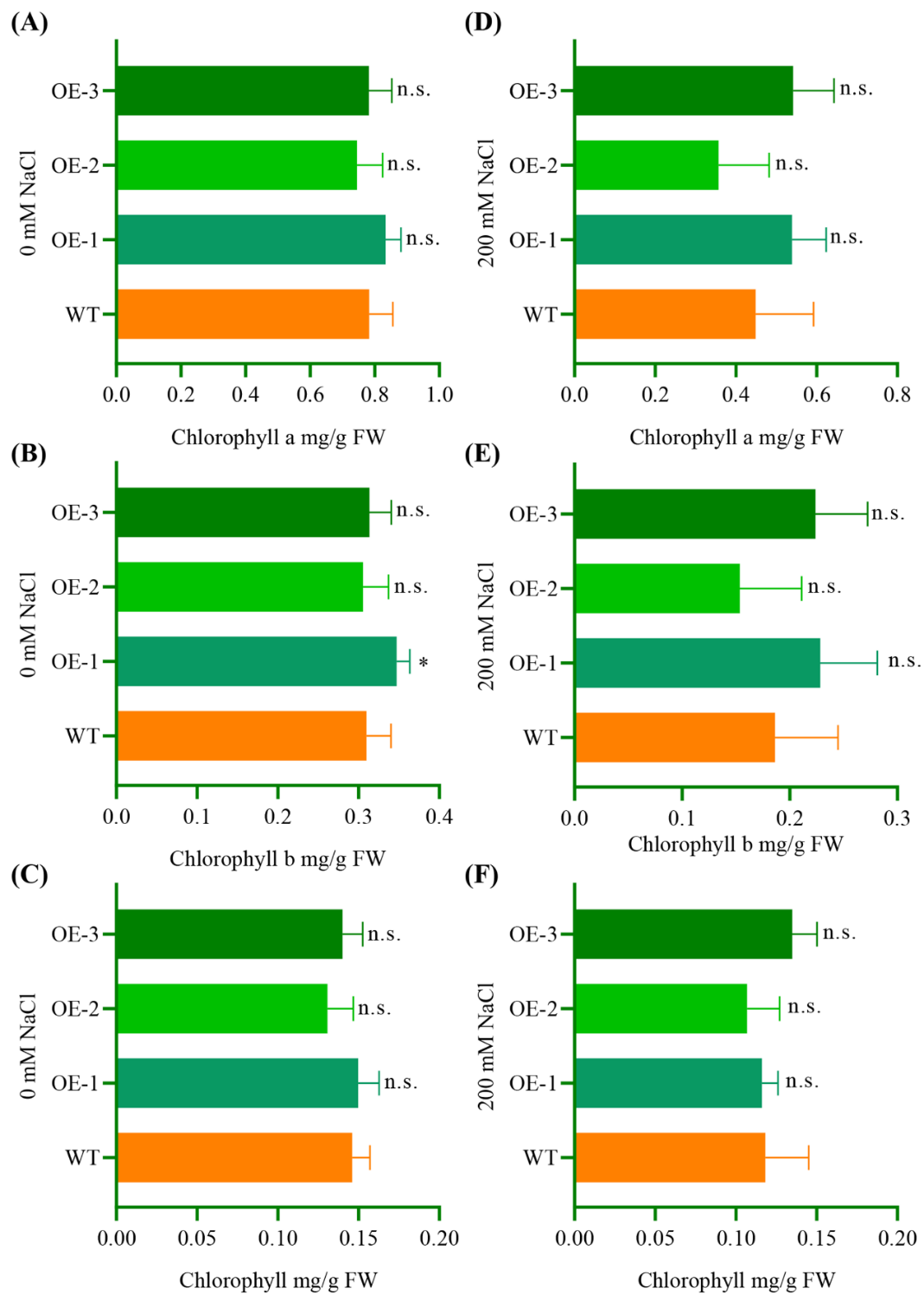


**Supplementary Figure 3. *NbCIPK25* overexpression does not affect chlorophyll content under salt treatment.**

**(A-F)** Chlorophyll a, Chlorophyll b and total Chlorophyll content under normal condition and 200 mM NaCl treatment in each genotype as indicated. Data represent means ± SD from three biological replicates, t-test used for statistics, ‘*’ *p* < 0.05, n.s.: not significant.
